# Tracing the evolution of polypharmacy and contraindicated drug‒drug interactions in people living with HIV in Belgium

**DOI:** 10.3389/fphar.2025.1632108

**Published:** 2025-09-22

**Authors:** Victoria Lopez Delhoulle, Li-Cécile Destordeur, Nathalie Maes, Karine Fombellida, Majdouline El Moussaoui, Gilles Darcis

**Affiliations:** ^1^ University of Liège, Liège, Belgium; ^2^ Biostatistics and Research Method Center, University Hospital of Liège, Liège, Belgium; ^3^ Infectious Diseases Department, Liège University Hospital of Liège, Liège, Belgium; ^4^ Internal general medicine, Liège University Hospital of Liège, Liège, Belgium

**Keywords:** polypharmacy, drug‒drug interactions, DDIs, antiretroviral, ARV, red-flag interactions, ageing, people living with HIV

## Abstract

**Background:**

People living with HIV (PWH) are more likely to develop comorbid conditions, which increases the likelihood of polypharmacy and potentially harmful drug–drug interactions (DDIs). As antiretroviral (ARV) therapies evolve, the nature and frequency of these interactions also change, highlighting the need for continued vigilance.

**Methods:**

We conducted a retrospective cohort study at the University Hospital of Liège (Belgium). We collected and analysed antiretroviral drugs (ARVs), comedications, and demographic and clinical data from 2017 to 2022. We used the University of Liverpool HIV drug interaction database to identify contraindicated red-flag interactions.

**Results:**

We observed a significant and continuous increase in the use of non-ARV medications in our cohort. Drug-drug interactions (DDIs) remained common and usually involved a boosted ARV regimen. The number of red-flag DDIs decreased over time after 2017 for several reasons including switching to unboosted ARV regimens. Topical steroids and proton pump inhibitors were the drugs most frequently involved in contraindicated DDIs among the comedications.

**Conclusion:**

Polypharmacy in people living with HIV (PWH) is a growing concern. Although the level of contraindicated drug-drug interactions (DDIs) has decreased over time, it remains a significant issue. Active monitoring and the implementation of alert systems can help clinicians mitigate the risk of such interactions.

## Introduction

By 2024, there were more than 40 million people living with HIV, of whom over 31 million had access to treatment. Antiretroviral (ARV) treatments have significantly increased the life expectancy of people living with HIV (PWH), transforming HIV into a chronic condition requiring lifelong management (Marcus et al., 2020; Gueler et al., 2017; Samji et al., 2013). ARVs target different stages of the HIV lifecycle. NRTIs (nucleoside or nucleotide reverse-transcriptase inhibitors) and NNRTIs (Non-nucleoside reverse-transcriptase inhibitors) block reverse transcriptase’s enzymatic function and prevent completion of synthesis of the double-stranded viral DNA. INSTIs (Integrase Strand Transfer Inhibitors) prevent viral DNA integration into the host genome. PIs (protease inhibitors) inhibit viral protease, blocking virus maturation, and are often combined with boosters (ritonavir or cobicistat) that inhibit CYP3A4 to increase drug levels.

Modelling studies project a significant increase in the median age of PWH receiving ARV therapy, accompanied by a growing burden of comorbidities (Smit et al., 2015; Smit et al., 2017).

People with HIV (PWH) are at greater risk of developing comorbidities and experiencing polypharmacy (defined as taking five or more non-antiretroviral (ARV) medications concurrently) earlier than the general population, which leads to increased costs for managing comorbidities (Guaraldi et al., 2011; Yang et al., 2019; Gimeno-Gracia et al., 2016; Li et al., 2025; Hopwood-Raja et al., 2025). For example, a Spanish study revealed that polypharmacy is more prevalent among older HIV-positive individuals than among similarly aged members of the general population. This report found that HIV-positive individuals were prescribed more central nervous system (CNS) drugs and anti-infectives (Gimeno-Gracia et al., 2016). In a Canadian cohort of people aged 65 and older living with HIV, more than half of the participants had polypharmacy (Hopwood-Raja et al., 2025). According to 2023 epidemiological data, more than half (51%) of people living with HIV in Belgium were aged 50 years or over. The average duration since diagnosis for PWH in follow-up care increased from 7 years in 2006 to 14 years in 2024 (Deblonde et al., 2024). This ageing population faces heightened vulnerability to chronic diseases, which leads to increased medication use and higher rates of polypharmacy. This subsequently increases in the risk of drug‒drug interactions (DDIs) (Back and Marzolini, 2020; Mazzitelli et al., 2024). In elderly HIV-positive patients, the duration of HIV infection is a stronger predictor of multimorbidity and polypharmacy than age alone (Guaraldi et al., 2018).

DDIs occur through mechanisms such as the inhibition or induction of liver enzymes (e.g., CYP3A4), changes in drug absorption in the digestive tract (e.g., chelation or pH modifications), and altered renal drug excretion. They are particularly expected when the drugs share metabolic pathways (e.g., CYP450 enzymes).

DDIs in individuals receiving HIV treatment can alter medication levels, causing toxicity, reduced efficacy, or resistance to ARV medications (Back and Marzolini, 2020; Nhean et al., 2021).

Given the many options for HIV therapy, selecting a regimen for an individual should be guided by factors such as virologic efficacy, toxicity, pill burden, dosing frequency, and the potential for drug‒drug interactions. For example, PWH treated with ritonavir or cobicistat boosted protease or integrase inhibitors experienced clinically relevant interactions more frequently than those on other regimens (de Oliveira Costa et al., 2023).

Currently, regimens including an integrase strand transfer inhibitor (INSTI) are the most commonly prescribed. Unboosted INSTIs are often favoured for various reasons, including a lower risk of DDIs (Back and Marzolini, 2020; Deutschmann et al., 2021; Lepik et al., 2022; Peng et al., 2024). In addition, some dual ARV therapies achieve therapeutic efficacy comparable to that of triple therapy and are recommended as switch or initial treatments. Switching standard triple therapy to dual therapy for individuals receiving antiretroviral treatment could also help reduce the risk of DDIs, as dolutegravir and lamivudine were among the regimens with the lowest rates of relevant potential DDIs in a recent report (de Oliveira Costa et al., 2023).

Although PWH are ageing, the proportion of individuals with potential DDIs could thus decrease over time with a switch to newer therapies that could reduce the number of DDIs. In addition, an alert system was implemented following previous alarming results showing an important number of DDIs in our centre in 2012 and 2016 (El Moussaoui et al., 2020).

The present study aimed to analyse the evolution of the prevalence and types of contraindicated drug-drug interactions (DDIs), also known as ‘red-flag’ DDIs, among PWH between 2017 and 2022 and to compare our findings with those of a previous study conducted at the University Hospital of Liège (2012–2016). ‘Red-flag interactions’ refer to severe, potentially life-threatening drug interactions that are absolutely contraindicated or require immediate intervention to avoid serious adverse effects.

## Methods

We conducted a retrospective longitudinal cohort study of individuals aged 18 years and over living with HIV and attended the University Hospital of Liège, Belgium, as outpatients, between 2017 and 2022. Participants who did not attend a medical consultation every year were excluded from the study, to enable annual data collection. All of the other participants were included in the study.

Demographic data included age, sex, ethnicity, and country of origin. Clinical data included weight, height, BMI, the date of the first positive HIV test result, the date of the first infectious disease consultation, the mode of transmission, and information on alcohol consumption status, smoking status, drug use, and concomitant medications. Biological data included the nadir CD4 count, the presence of other conditions (including hepatitis B or C), and HIV type (1 or 2). Data on non-ARV medications were collected at each visit to an infectious disease specialist and categorized according to the Belgian Center for Pharmacotherapeutic Information (CBIP) standards. The CBIP is an official drug evaluation organization that provides a standardized therapeutic classification system.

We used the Liverpool Interaction Database to identify contraindicated (red flag) drug interactions (Liverpool HIV Interactions, 2023). The charts used in this study were available on the Liverpool Interaction Checker and were last revised on 31 May 2023. This study focused exclusively on contraindicated drug-drug interactions (DDIs) between antiretroviral (ARV) and non-ARV medications.

To enable comparison with the years 2012 and 2016 (El Moussaoui et al., 2020), participants were selected who had been followed up in both 2012 and 2016, as well as between 2017 and 2022. Red-flag interactions for these participants were analysed using the same charts, which were available on the Liverpool Interaction Checker and revised on 31 May 2023.

### Statistical analysis

Quantitative variables are presented as the means and standard deviations (means ± SD) or medians and interquartile ranges (Median (Q1–Q3)). Qualitative variables are summarized using frequency tables (counts and percentages). The temporal evolution of the number of patients with DDIs was analysed using generalized estimating equation (GEE) models for repeated measures. GEE are used to estimate the parameters of a generalized linear model when there is a possible unmeasured correlation between observations from different time points. In other words, GEE was used to account for potential correlations between repeated measurements within participants over time. This method provides population-averaged effect estimates that are robust to within-subject dependencies, making it suitable for analyzing longitudinal data where observations may not be fully independent. A multiple GEE model was employed to investigate the factors influencing the risk of DDIs. Analyses were performed using the maximum available data, and missing values were not imputed. The results are presented as p-values, adjusted odds ratios (ORs), and 95% confidence intervals. Results were considered significant at the 5% level (p < 0.05). All analyses were conducted using SAS software (version 9.4).

### Ethical considerations

Approval for the study protocol was obtained from the local ethics review committee (Comité d’Ethique Hospitalo-Facultaire Universitaire de Liège; reference number 2023–261). The participants were informed of the data collected by their treating physician and could object to further collection of clinical data. All participants included were assigned unique identification numbers to anonymize the data and protect their confidentiality. The need to obtain individual consent was waived because of the retrospective nature of the study and the anonymization of the data. All methods were carried out in accordance with relevant guidelines and regulations.

## Results

### Participant characteristics

We included 812 participants aged 18–80 years in 2017 who had at least one consultation per year from 2017 to 2022 (Supplementary Table S1). The average age was 42.8 ± 11.2 years. Consequently, the average age in 2022 was 47.8 years. Thirty-five percent of the participants were African women with heterosexual transmission, and 28.5% were white men with homosexual/bisexual transmission. A detailed description of the cohort is provided in Supplementary Table S1.

### ARV regimen used

The most common ARV regimen in 2022 was 2NRTIs + an INSTI (33%, 267/812), followed by 1NRTI + an INSTI (27%, 217/812) and 2NRTIs + an NNRTI (14%, 113/812) (Supplementary Table S2). In 2017, 64% of participants were on an INSTI-based regimen (522/812). This percentage increased to 82% in 2022 (668/812). In 2017, 3.2% (26/812) of the participants were on a dual therapy regimen. This percentage increased to 35% (282/812) by 2022. Conversely, the percentage of participants on triple therapy decreased from 89% (726/812) in 2017 to 62% (501/812) in 2022. Additionally, the use of regimens involving four or more ARV medications decreased from 4.6% (37/812) in 2017 to 3.3% (27/812) in 2022.

The number of participants who received boosters significantly decreased between 2017 and 2022 (p < 0.0001) (Supplementary Table S3). In 2017, 21% (171/812) of the participants were receiving treatment that included cobicistat, and 11% (91/812) were on a regimen including ritonavir. In 2022, the percentage of participants decreased to 15% (121/812) and 2% (19/812) for cobicistat and ritonavir, respectively (Supplementary Tables S2, S3).

### Comedications and polypharmacy

The number of non-ARV comedications used significantly increased over time (p < 0.0001) (Table 1). The percentage of participants receiving at least one comedication increased from 81.7% in 2017 to 88.6% in 2022 (p < 0.0001). Additionally, the percentage of participants with polypharmacy increased from 20.7% to 28.9% during this period (p < 0.0001) (Table 1).

**TABLE 1:** Number of non-ARV comedications used and drug‒drug interactions in participants with at least one consultation per year between 2017 and 2022 at the University Hospital of Liège, Belgium, including data from 543 participants collected in 2012 and 2016.

Variables	N = 543 participants who were followed up in 2012 and 2016 and had at least one consultation per year from 2017 to 2022			N = 812 participants who had at least one consultation per year from 2017 to 2022						
	2012	2016	p value (2012–2016)	2017	2018	2019	2020	2021	2022	p value (2017–2022)
Number of participants	543	543		812	812	812	812	812	812	
Number of participants with no comedication use (%)	114 (21.0)	73 (13.4)		149 (18.3)	124 (15.3)	110 (13.6)	121 (14.9)	101 (12.4)	93 (11.4)	
Number of participants with 1 or more comedications (%)	429 (79.0)	470 (86.6)	<0.0001^a^	663 (81.7)	688 (84.7)	702 (86.4)	691 (85.1)	711 (87.6)	719 (88.6)	<0.0001^b^
1–4	*335*	*359*		*495*	*509*	*516*	*480*	*481*	*484*	
≥5	*94*	*111*		*168*	*179*	*186*	*211*	*230*	*235*	
Mean ± SD	2.3 ± 2.3	2.9 ± 2.7	0.0005^c^	2.7 ± 2.8	2.9 ± 2.9	3.1 ± 3.1	3.2 ± 3.1	3.4 ± 3.2	3.5 ± 3.2	<0.0001^c^
Median (Q1 – Q3)	2 (1–3)	2 (1–4)		2 (1–4)	2 (1–4)	2 (1–4)	2 (1–5)	3 (1–5)	3 (1–5)	
Min – Max	0–15	0–19		0–17	0–18	0–23	0–21	0–23	0–20	
Number of participants with DDIs (%)	30 (5.5)	33 (6.1)	0.61^a^	31 (3.8)	29 (3.6)	20 (2.5)	19 (2.3)	24 (3.0)	18 (2.2)	0.064^b^
Number of DDIs per participant										
0	513 (94.5)	510 (93.9)		781 (96.2)	783 (96.4)	792 (97.5)	793 (97.7)	788 (97.0)	794 (97.8)	
1	12 (2.2)	19 (3.5)		24 (3.0)	20 (2.5)	12 (1.5)	13 (1.6)	15 (1.9)	13 (1.6)	
2	13 (2.4)	7 (1.3)		2 (0.2)	7 (0.9)	6 (0.7)	4 (0.5)	8 (1.0)	5 (0.6)	
3	1 (0.2)	2 (0.4)		2 (0.2)	2 (0.2)	1 (0.1)	2 (0.2)	0 (0.0)	0 (0.0)	
4	3 (0.5)	4 (0.7)		3 (0.4)	0 (0.0)	1 (0.1)	0 (0.0)	0 (0.0)	0 (0.0)	
5	1 (0.2)	0 (0.0)		0 (0.0)	0 (0.0)	0 (0.0)	0 (0.0)	1 (0.1)	0 (0.0)	
6	0 (0.0)	1 (0.2)		0 (0.0)	0 (0.0)	0 (0.0)	0 (0.0)	0 (0.0)	0 (0.0)	
Total number of DDIs	58 (N = 543)	61 (N = 543)	-	46 (N = 812)	40 (N = 812)	31 (N = 812)	27 (N = 812)	36 (N = 812)	23 (N = 812)	0.044^d^

^a^ McNemar test for repeated measurements.

^b^ Generalized estimating equation (GEE) model.

^c^ linear mixed model.

^d^ linear model.

We also conducted an analysis of a subgroup of patients (543 participants) who were followed up in 2012 and 2016, as well as between 2017 and 2022 (see Supplementary Table S4). This revealed a statistically significant increase in both the prevalence and the number of non-ARV comedications used by the participants between 2012 and 2022. The percentage of participants receiving at least one non-ARV comedication increased during this period, rising from 79% in 2012 to 92.6% in 2022 (Supplementary Table S4). Furthermore, the average number of non-ARV comedications used by each participant showed a similar upward trend, rising from 2.3 in 2012 to 3.9 in 2022 (Supplementary Table S4).

### Red-flag drug‒drug interactions

The risk of red-flag DDIs remained stable between 2012 and 2016 (Table 1; Supplementary Table S4). The percentage of participants with at least one red-flag DDI decreased from 3.8% in 2017 to 2.2% in 2022, indicating a statistically nonsignificant trend towards reduction (p = 0.064) (Table 1). However, the total number of DDIs (some participants had more than one red-flag DDI) decreased by half, from 46 in 2017 to 23 in 2022, indicating a statistically significant reduction (p = 0.044) (Table 1).

We also conducted a similar analysis on a subgroup of patients (543 participants) who were followed up in 2012 and 2016, as well as between 2017 and 2022 (see Supplementary Table S4). From 2017 to 2022, we observed a statistically significant linear decrease in the risk of DDIs per participant (p = 0.0017) (Table 2), while the number of comedications used was increasing (p < 0.0001).

**TABLE 2:** Evolution of the number of comedications used and DDIs over time, 2017–2022 (GEE model).

Variables	Coeff ± SD	p value
Intercept	−3.8 ± 0.20	-
After 2017 (1 = Yes)	0.97 ± 0.59	0.097
Time (in years since 2012) since 2017	−0.24 ± 0.078	0.0017
Number of comedications used (non-ARV medications)	0.26 ± 0.029	<0.0001

### Predictors of red-flag drug‒drug interactions

**FIGURE 1:**
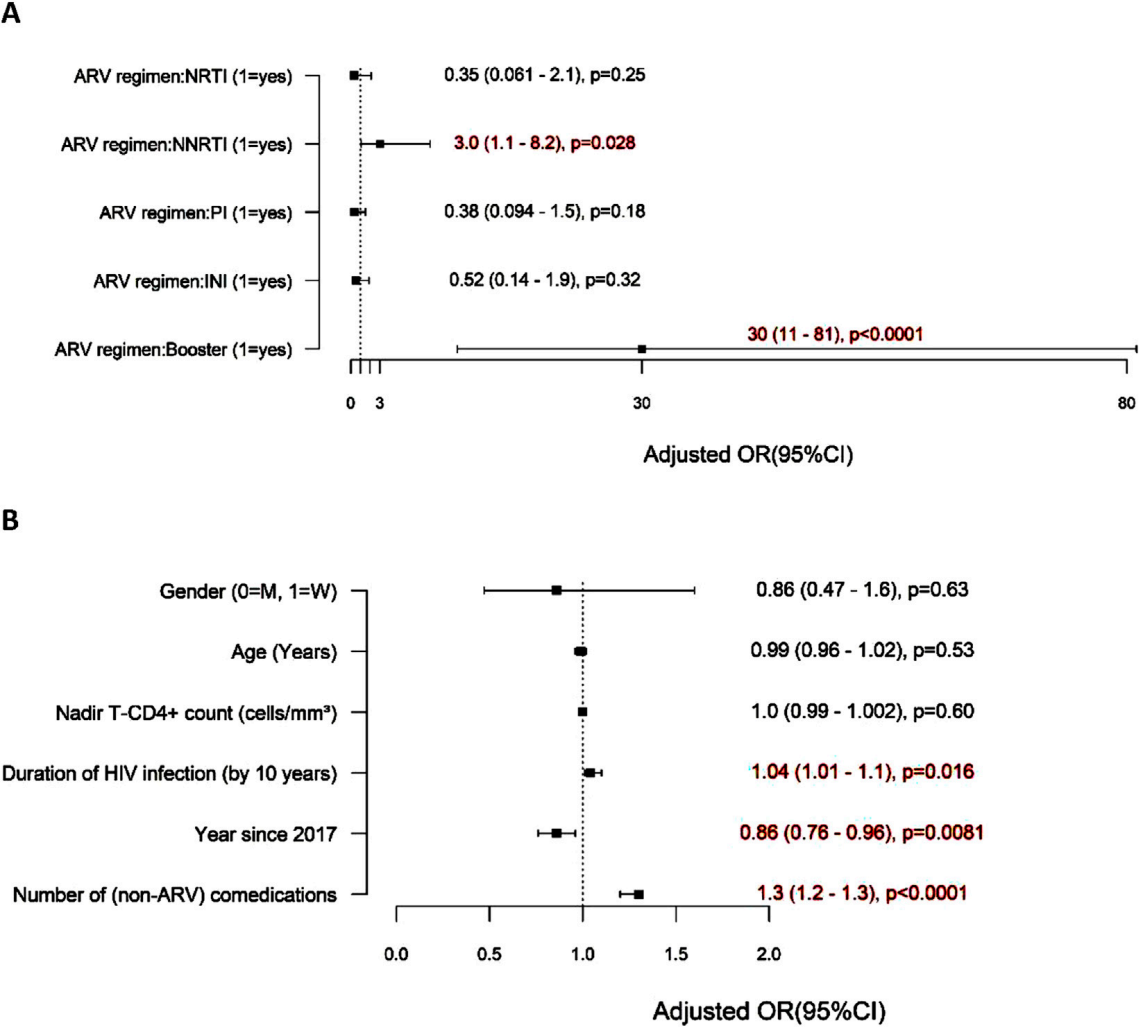
Factors associated with the risk of red-flag DDIs. **(A)** ARV regimen associated with DDIs. **(B)** Other factors associated with DDIs.

The type of ARV regimen plays a major role in DDIs (Figure 1A). Specifically, participants with boosted regimens were 30 times more likely to experience a DDI than those without boosted regimens are (95% CI: 11 to 81, p < 0.0001). Similarly, patients on NNRTIs were three times more likely to experience a DDI than are those not on NNRTIs (95% CI: 1.1 to 8.2, p = 0.028).

The risk factors for DDIs include several variables, notably the number of non-ARV comedications used, which significantly increases the risk of DDIs (Figure 1B). For each additional non-ARV comedication used, the risk of DDIs increased by 30% (95% CI: 1.2 to 1.3, p < 0.0001). The duration of HIV infection was also a contributing factor, as each 10-year interval since the first known positive HIV test was associated with a 4% increase in the risk of DDIs (95% CI: 1.01 to 1.1, p = 0.016). Interestingly, the risk of red-flag DDIs decreased with time after 2017 (by 14% annually) (95% CI: 4%–24%, p = 0.0081). Age, sex, and the nadir CD4 count were not found to impact the risk of DDIs.

These findings suggest that both the number of comedications used and the type of ARV regimen significantly influence the risk of DDIs in patients with HIV.

### ARV drugs implicated in DDIs

In 2017, eleven ARV drugs were associated with DDIs (Table 3). The ARV regimen most frequently used was EVG/c/F/TAF, accounting for 28% (13/46) of DDIs. Boosted elvitegravir (EVG/c/F/TAF + EVG/c/F/TDF) accounted for 37% ((13 + 4)/46; Table 3).

**TABLE 3:** ARV and non-ARV drugs involved in red-flag DDIs (N = 812 participants with at least one consultation per year from 2017 to 2022).

Treatment			Year					
			2017	2018	2019	2020	2021	2022
Antiretroviral								
NNRTI	RPV	Rilpivirine	1					
NRTI + NRTI	F/TAF	Emtricitabine/Tenofovir alafenamide	1	1	1			
NNRTI + NRTI + NRTI	EFV/F/TDF	Efavirenz/Emtricitabine/Tenofovir disoproxil fumarate		1				
	RPV/F/TDF	Rilpivirine/Emtricitabine/Tenofovir disoproxil fumarate	1	1	2	2	3	3
	RPV/F/TAF	Rilpivirine/Emtricitabine/Tenofovir alafenamide	1				2	1
INSTI + NNRTI	DTG/RPV	Dolutegravir/Rilpivirine				1	1	1
PI	ATV	Atazanavir	3	2	1	2	1	
PI + Booster	DRV/r	Darunavir/Ritonavir	8	14	10	8	12	8
	LPV/r	Lopinavir/Ritonavir	7					
	ATV/r	Atazanavir/Ritonavir	2		2	2	2	
	DRV/c	Darunavir/Cobicistat	5	2	3	4	3	2
PI + Booster + NRTI + NRTI	DRV/c/F/TAF	Darunavir/Cobicistat/Emtricitabine/Tenofovir alafenamide			1	1	3	2
INSTI + booster + NRTI + NRTI	EVG/c/F/TDF	Elvitegravir/Cobicistat/Emtricitabine/Tenofovir disoproxil fumarate	4	1	1			
	EVG/c/F/TAF	Elvitegravir/Cobicistat/Emtricitabine/Tenofovir alafenamide	13	18	10	7	9	6
Non-ARV								
Corticosteroids	Budesonide			1				
	Budesonide (topical)		9	5	8	5	6	4
	Fluticasone (topical)		5	2	2	3	4	3
	Mometasone (topical)		2	6	4	3	4	3
Proton pump inhibitors (PPI)	Omeprazole		1	1	1	2	1	
	Pantoprazole		4	2	2	1	3	3
	Esomeprazole		1			2	3	2
Antiaggregants	Clopidogrel		3	1	2	1	1	
	Ticagrelor		1					
Anticoagulants	Apixaban						1	2
	Rivaroxaban		1	1			4	2
NSAIDs	Piroxicam		1	1				
Lipid lowering agent	Simvastatine		6	6	2	3	2	1
	Red yeast rice		4	2	1			
Calcium antagonist	Lercanidipine		5	5	7	6	6	3
Antipsychotic	Haloperidol		1					
	Quetiapine				1	1	1	
Antiepileptic	Carbamazepine		1	4	1			
	Flecainide		1	1				
Antiemetic	Domperidone			1				
Birth control pill	Birth control pill			1				
Total number of DDIs			46	40	31	27	36	23

Darunavir, in combination with either cobicistat or ritonavir, accounted for another 28% (8 + 5/46) of DDIs, whereas boosted lopinavir (LPV/r) accounted for 15% (7/46) of DDIs. Boosters were involved in the majority of DDIs, with cobicistat present in 48% (22/46) and ritonavir present in 37% (17/46) of cases.

By 2022, eight ARV regimens were implicated in DDIs (Table 3). Boosted darunavir accounted for 43% (8 + 2/23) of DDIs, and EVG/c/F/TAF was involved in 26% (6/23) of DDIs (Table 3). Boosters continued to play a major role in 2022, with cobicistat being implicated in 43% (10/23) and ritonavir in 35% (8/23) of contraindicated DDIs. Rilpivirine-based regimens were also associated with a high proportion of red-flag DDIs, as they were involved in the remaining 5/23 red-flag DDIs in 2022, all of which involved proton pump inhibitors (PPIs), as described below.

### Non-ARV drugs implicated in DDIs

Table 3 presents the detailed non-ARV drugs involved in DDIs, while Supplementary Table S5 presents the classes of non-ARV drugs involved in DDIs.

In 2017, the majority of non-ARV-related DDIs were caused by cardiovascular drugs (26%, 12/46; Supplementary Table S5), including lercanidipine, which was used as an antihypertensive calcium channel blocker, followed by respiratory drugs (inhaled corticosteroids) (20%, 9/46; see Supplementary Table S5), proton pump inhibitors (PPIs, 13%, 6/46), and antiplatelets/anticoagulants (11%, 5/46) (Supplementary Table S5). Topical corticosteroids, which can be used not only as respiratory drugs but also to treat inflammatory bowel disease (IBD), dermatological disorders and ear, nose and throat conditions were extremely important, contributing to 35% (16/46) of DDIs (Table 3).

By 2022, PPIs were responsible for 22% (5/23; Supplementary Table S5) of DDIs, whereas cardiovascular drugs, anticoagulants, and respiratory drugs (inhaled corticosteroids) each accounted for 17% (4/23) of DDIs (Supplementary Table S5). Topical corticosteroids were implicated in 43% (10/23) of DDIs in 2022 (Table 3).

Taken together, these findings suggest that boosters remain the primary issue associated with DDIs, particularly when DDIs are combined with (mostly topical) steroids, antihypertensive drugs such as lercanidipine and antiplatelets/anticoagulants. In 2022, the use of rilpivirine (alone or in combination) with PPIs remained associated with a large proportion of red-flag DDIs (Table 3).

## Discussion

This longitudinal retrospective study assessed the evolution and type of red-flag DDIs in a cohort of people living with HIV in Liège, Belgium, from 2017 to 2022. We also compared our findings with the results of previously published studies that analysed DDIs in 2012 and 2016 (El Moussaoui et al., 2020). Our results revealed a significant increase in the number of non-ARV comedications used, which has been identified as a risk factor for DDIs, as reported by other groups (Ok et al., 2020; Ruellan et al., 2021; Altunal et al., 2023). This is not surprising, given that the average age of PWH is increasing, both in our cohort and globally. The proportion of participants with polypharmacy increased significantly in our study, rising from 20.7% to 28.9% between 2017 and 2022. Polypharmacy is undoubtedly a growing issue that needs to be addressed (De Bellis et al., 2025), as polypharmacy has been linked to non-adherence, adverse drug events, falls, opioid overdoses and increased complexity of medical regimens, thereby contributing to inappropriate prescribing (Justice et al., 2021).

Despite the substantial increase in comedications used, we observed a continuous reduction in the total number of DDIs since 2017, which contrasts with the findings of our previous reports from 2012 to 2016 (El Moussaoui et al., 2020). A decline in red-flag and/or orange-flag DDIs has also been documented in other studies (Deutschmann et al., 2021; Lepik et al., 2022). These reductions were mostly attributed to a shift from PI-based regimens to INSTI-based regimens. The decrease in red-flag DDIs in our study was related to multiple factors.

Firstly, there has been a shift from higher DDI-risk ART, mostly boosted regimens, to those with lower risk, particularly regarding the widespread usage of unboosted INSTIs, both in triple and dual therapy. The large reduction in the use of boosted regimens is a key factor associated with the reduction in DDIs, as a booster is associated with a 30-fold greater risk of red-flag DDIs. We thus confirmed other results showing a strong negative impact of a booster on the risk of DDIs (de Oliveira Costa et al., 2023; Altunal et al., 2023; Jakeman et al., 2017; Peng et al., 2024). In particular, boosted elvitegravir was implicated in 37% of DDIs in 2017 and 26% of DDIs in 2022 in our study, a score largely driven by its red-flag interaction with topical steroids and lercanidipine. Bictegravir- and dolutegravir-based regimens are now preferred because of their higher resistance barrier and better profile considering DDIs.

Second, the simplification of antiretroviral therapy regimens may contribute to the observed trend, with a growing usage of dual therapy and a shift away from more complex treatment combinations (Ruellan et al., 2021). We confirmed that the combination of NRTI/INSTI (3TC/DTG), which has been widely used in our centre and globally in recent years, is safe in terms of the risk of DDIs. Nevertheless, NNRTI/INSTI (RPV/DTG) dual therapy was associated with a higher risk of DDI, particularly when associated with proton pump inhibitors (Back and Marzolini, 2020; Hodge et al., 1999; Livio and Marzolini, 2019).

Finally, following our previous reports from 2012 to 2016, we implemented an alert system to warn clinicians that concurrent prescription may be inappropriate. This alert system is based on the University of Liverpool HIV drug interaction database. We regularly run this program that allows us to identify contraindicated interactions between ARVs and other medications listed in the electronic medical records. Clinicians are then notified in the event of contraindicated interactions. This system has probably contributed to changing clinicians’ habits and reducing the number of contraindicated interactions. However, this effect is difficult to isolate from other factors involved in reducing these interactions.

One of the identified risk factors for DDIs in our study was the duration of HIV infection rather than age itself. This may reflect an earlier onset of multimorbidity and subsequent polypharmacy in PWH, a phenomenon well known as premature ageing (Guaraldi et al., 2011; Gimeno-Gracia et al., 2016; Guaraldi et al., 2018).

In terms of comedications, topical corticosteroids emerged as the most frequently implicated non-ARV drugs in DDIs, accounting for almost half of the red-flag DDIs in 2022. This finding is consistent with previous studies, which have identified corticosteroids as one of the non-ARV drugs most frequently implicated in DDIs (de Oliveira Costa et al., 2023; López-Centeno et al., 2020). These medications mostly interact with boosted ARV medications, which inhibit CYP3A4, thereby increasing the risk of Cushing’s syndrome and adrenal insufficiency. Although the systemic absorption of topical corticosteroids is usually low, it can sometimes be significant, particularly when high-potency formulations are used or applied to large areas of skin. In such cases, the risk of interaction with pharmacokinetic boosters may become relevant. This risk could be mitigated by the prescription of alternative corticosteroids, such as beclomethasone (Liverpool HIV Interactions, 2023; EACS, 2023; World Health Orga nization, 2021). If coadministered, dose reduction of the glucocorticoid should be considered with close monitoring of local and systemic effects (Liverpool HIV Interactions, 2023).

The proportion of DDIs involving proton pump inhibitors (PPIs) and RPV remained high and fairly stable over time, accounting for almost a quarter of the red-flag DDIs in 2022. PPIs are frequently well-known causes of DDIs, as shown in other studies (de Oliveira Costa et al., 2023; Deutschmann et al., 2021; Steulet et al., 2024). Coadministration may significantly decrease the plasma concentration of rilpivirine. Indeed, PPIs reduce gastric acid secretion and thereby increase gastric pH, which can substantially impair the absorption of certain antiretroviral drugs that require an acidic environment for optimal bioavailability—such as rilpivirine and atazanavir. In the case of rilpivirine, coadministration with PPIs can lead to significantly reduced plasma concentrations, potentially compromising virologic suppression and increasing the risk of treatment failure. Alternatives include switching to intramuscular RPV or to another ARV unaffected by gastric pH (Liverpool HIV Interactions, 2023; EACS, 2023).

Cardiovascular and anti-haemostatic drugs each accounted for 17% of the DDIs in our study, a finding frequently reported in the literature (de Oliveira Costa et al., 2023; Lepik et al., 2022; López-Centeno et al., 2020). The molecules lercanidipine, clopidogrel and rivaroxaban were among the most frequently involved. PIs and boosters significantly increase the risk of adverse reactions by increasing the plasma concentrations of some of these drugs, including some statins, potentially leading to serious outcomes such as rhabdomyolysis. They also increased the risk of bleeding when taken alongside anticoagulants or clopidogrel. The clinical effect of DDIs between antiplatelets and antiretroviral therapy (ART) on bleeding, thrombosis, and other major adverse cardiovascular events (MACE) remains unclear (Matsikas et al., 2025). Regarding antihypertensive drugs, boosters further amplified plasma lercanidipine levels. Alternative antihypertensives should be considered to prevent these potentially severe interactions (Back and Marzolini, 2020; Nhean et al., 2021; Liverpool HIV Interactions, 2023; EACS, 2023).

PWH are particularly vulnerable to mental health conditions, which often necessitate the use of neuroleptics. According to the literature, neuroleptics are commonly prescribed to PWH (Deutschmann et al., 2021). We indeed identified specific DDIs involving haloperidol and quetiapine. In particular, quetiapine is known to be involved in several DDIs and, together with other atypical antipsychotics, is listed by the EACS guidelines as one of the top medications to be avoided and not prescribed to elderly individuals with HIV in certain conditions (Liverpool HIV Interactions, 2023; EACS, 2023).

Of course, the issue of drug-drug interactions (DDIs) is not limited to people living with HIV (PWH). Antiviral drugs are also used in other contexts, such as for treating hepatitis B and C, and for treating patients with SARS-CoV-2 (Hodge et al., 1999; Livio and Marzolini, 2019). In the context of SARS-CoV-2 infection, the most frequent DDIs involve nirmatrelvir/ritonavir (Hodge et al., 1999), which is consistent with our results. Therefore, SARS-CoV-2 infection in PWH is associated with an increased risk of DDIs, necessitating vigilance and a personalised therapeutic approach (Conti et al., 2024; Hendrick et al., 2024).

Our findings should be interpreted in light of several limitations. Patients often fail to disclose all over-the-counter medications or dietary supplements used, and short-term treatments such as antibiotics are difficult to track accurately, potentially introducing bias into the study. Additionally, we did not analyse interactions between ARV medications themselves.

In conclusion, we showed a significant and continuous increase in the number of non-ARV comedications used in our cohort. The risk of DDIs remains closely linked to the number of non-ARV comedications used, although the number of red-flag DDIs decreased with time, a reduction mostly triggered by the switch to an unboosted ARV regimen. Topical steroids, when associated with a booster, and PPIs, when associated with RPV, are the most frequent comedications associated with DDIs. Our findings emphasize the importance of ongoing vigilance in identifying and managing DDIs in PWH. Active monitoring, along with the implementation of alert systems, can help healthcare providers avoid potentially harmful interactions. Favoring ARVs with a low risk of interaction could help reduce the risk of interaction. In addition, it is crucial to raise awareness among healthcare providers, including not only infectious disease specialists, but also general practitioners. Patients themselves need to be made aware of the risk of drug interactions, and how to ensure that they do not occur when a new drug is prescribed or taken without any prescription. Distribution points also have a role to play in preventing drug interactions, especially where prescription-free medication access exists.

## Data Availability

The raw data supporting the conclusions of this article will be made available by the authors, without undue reservation.

## Abbreviations

NRTI, Nucleoside Reverse Transcriptase Inhibitor; NNRTI, Non-Nucleoside Reverse Transcriptase Inhibitor; INSTI, Integrase Strand Transfer Inhibitor; PI, Protease Inhibitor; Booster, Pharmacokinetic Enhancer; EFV, Efavirenz *(NNRTI)*; RPV, Rilpivirine *(NNRTI)*; FTC, Emtricitabine *(NRTI)*; TDF, Tenofovir disoproxil fumarate *(NRTI)*; TAF, Tenofovir alafenamide *(NRTI)*; DTG, Dolutegravir *(INSTI)*; EVG, Elvitegravir *(INSTI)*; ATV, Atazanavir *(PI)*; DRV, Darunavir *(PI)*; LPV, Lopinavir *(PI)*; r, Ritonavir *(booster)*; c, Cobicistat *(booster*).
