# Supplementary material for: Tracing the evolution of polypharmacy and contraindicated drug‒drug interactions in people living with HIV in Belgium
Source: Front Pharmacol. 2025 Sep 22;16:1632108. doi: 10.3389/fphar.2025.1632108 (PMC12497831; doi:10.3389/fphar.2025.1632108)
Supplement: Supplementary file 1 [file Table1.docx]

**Supplementary**

**Table S1: Description of the sample 2017-2022 (number of participants = 812)**

|  | Number of non-missing values | Results |
| --- | --- | --- |
| Sex, Men | 812 | 435 (53.6) |
| Age* (Years) | 812 | 42.8 ± 11.2 |
| Weight (kg) | 812 | 73.9 ± 14.2 |
| Height (cm) | 812 | 170 ± 9 |
| BMI (kg/m²) | 812 | 25.6 ± 4.7 |
|  |  |  |
| Tabacco consumption | 789 |  |
| Never |  | 506 (64.1) |
| Stop ≥1 year |  | 77 (9.8) |
| Stop <1 year |  | 21 (2.7) |
| Yes |  | 185 (23.4) |
|  |  |  |
| Alcohol consumption | 711 |  |
| Never |  | 223 (31.4) |
| Stop ≥1 year |  | 10 (1.4) |
| Stop <1 year |  | 8 (1.1) |
| Yes (Occasionally or <4 glasses) |  | 460 (64.7) |
| Yes (≥4 glasses) |  | 10 (1.4) |
|  |  |  |
| Ethnicity | 812 |  |
| White |  | 357 (44.0) |
| African |  | 423 (52.1) |
| Other |  | 32 (3.9) |
|  |  |  |
| Contamination mode | 785 |  |
| Heterosexual (with or without use of drugs) |  | 495 (63.1) |
| Homo/bisexual (with or without use of drugs) |  | 244 (31.1) |
| Non-haemophilic transfusion |  | 17 (2.2) |
| Mother-to-child transfusion |  | 14 (1.8) |
| Drug use |  | 5 (0.6) |
| Others |  | 10 (1.2) |
|  |  |  |
| African women with heterosexual transmission | 785 | 275 (35.0) |
| White men homo/bisexual transmission |  | 224 (28.5) |
| Others |  | 286 (36.4) |
|  |  |  |
| Hepatitis immunization and infection | 812 | 121 (14.9) |
| Hepatitis A | 632 |  |
| Non-immunized |  | 141 (22.3) |
| Immunized |  | 491 (77.7) |
| Hepatitis B | 810 |  |
| Non-immunized |  | 271 (33.5) |
| Immunized |  | 416 (51.4) |
| Chronic infection |  | 48 (5.9) |
| Isolated HBc antibodies isolated |  | 75 (9.3) |
| Hepatitis C | 812 |  |
| Non-infected |  | 781 (96.2) |
| Acute infection |  | 0 (0.0) |
| Chronic infection |  | 2 (2.5) |
| Cured |  | 11 (1.4) |
|  |  |  |
| HIV2 | 812 | 5 (0.6) |
|  |  |  |
| CD4 nadir (number of cells/mm³) | 812 | 276 (147 – 424) |
| Number of years* since the first HIV test known | 812 | 126 (61 – 196) |

*The results are expressed using mean ± standard deviation, median (Q1 – Q3), or Number (%).*

** At first January 2017*

**Table S2 : ARV regimen classes in participants from 2017 to 2022.**

| Ongoing treatment (as reported during the first consultation of the considered year) | 2017  N | 2018  N | 2019  N | 2020  N | 2021  N | 2022  N |
| --- | --- | --- | --- | --- | --- | --- |
| 2NRTI | 1 | . | . | . | . | . |
| 2NRTI + INSTI | 303 | 328 | 352 | 348 | 299 | 267 |
| 2NRTI + INSTI + Cobicistat | 131 | 157 | 143 | 128 | 96 | 66 |
| 2NRTI + INSTI + entry inhibitor | 1 | 1 | 1 | 1 | 1 | 1 |
| 2NRTI + NNRTI | 180 | 160 | 146 | 132 | 125 | 113 |
| 2NRTI + NNRTI + INSTI | 4 | 3 | 3 | 3 | 3 | 3 |
| 2NRTI + NNRTI + PI + INSTI + Cobicistat | . | 1 | 1 | 1 | 1 | 1 |
| 2NRTI + NNRTI + PI + INSTI + Ritonavir | 2 | 1 | 1 | 1 | 1 | 1 |
| 2NRTI + PI | 2 | . | . | . | . | . |
| 2NRTI + PI + Cobicistat | 8 | 14 | 23 | 25 | 24 | 20 |
| 2NRTI + PI + INSTI + Cobicistat | 9 | 12 | 14 | 16 | 16 | 15 |
| 2NRTI + PI + INSTI + entry inhibitor + Ritonavir | 2 | 2 | . | . | . | . |
| 2NRTI + PI + INSTI + Ritonavir | 4 | 2 | 1 | . | . | . |
| 2NRTI + PI + Ritonavir | 63 | 40 | 35 | 23 | 21 | 8 |
| 3NRTI | 3 | 2 | 2 | 2 | . | . |
| 3NRTI + INSTI | 7 | 7 | 6 | 4 | 2 | 2 |
| 3NRTI + NNRTI | 1 | 1 | . | . | . | . |
| 3NRTI + PI + Cobicistat | 1 | 1 | 1 | . | . | . |
| 3NRTI + PI + INSTI + Cobicistat | . | 1 | 2 | 2 | 2 | 2 |
| 3NRTI + PI + Ritonavir | 1 | 1 | 1 | 1 | 1 | . |
| NNRTI + INSTI | 15 | 19 | 28 | 35 | 32 | 58 |
| NNRTI + INSTI + entry inhibitor | 3 | 1 | 1 | 1 | 1 | 1 |
| NNRTI + PI + INSTI + Cobicistat | 10 | 9 | 9 | 9 | 8 | 8 |
| NNRTI + PI + INSTI + Ritonavir | 9 | 9 | 7 | 7 | 7 | 7 |
| NNRTI + PI + Ritonavir | 1 | 1 | . | . | . | . |
| NRTI + INSTI | 1 | 4 | 8 | 51 | 152 | 217 |
| NRTI + NNRTI + INSTI | 4 | 3 | 4 | 4 | 7 | 7 |
| NRTI + NNRTI + INSTI + entry inhibitor | 1 | 1 | 1 | 1 | . | . |
| NRTI + NNRTI + INSTI + entry inhibitor + Cobicistat | 1 | . | . | . | . | . |
| NRTI + NNRTI + PI + Cobicistat | 1 | . | . | . | . | . |
| NRTI + NNRTI + PI + INSTI + Ritonavir | . | . | . | . | 1 | 1 |
| NRTI + NNRTI + PI + Ritonavir | 4 | 2 | 3 | 3 | 1 | 1 |
| NRTI + NNRTI+ PI + INSTI + Ritonavir | 2 | 2 | 2 | 2 | . | . |
| NRTI + PI + INSTI + Cobicistat | 2 | 4 | 3 | 2 | 2 | 2 |
| NRTI + PI + INSTI + entry inhibitor + Cobicistat | 1 | 1 | 1 | 1 | 1 | 1 |
| NRTI + PI + INSTI + Ritonavir | 3 | . | . | . | 1 | 2 |
| NRTI + PI + Ritonavir | 1 | . | . | . | . | . |
| PI + Cobicistat | 2 | . | . | . | . | . |
| PI + INSTI + Cobicistat | 6 | 8 | 7 | 6 | 6 | 6 |
| PI + INSTI + Ritonavir | 1 | 1 | 1 | . | . | . |
| Other or not known | 21 | 13 | 5 | 3 | 1 | 2 |
| Total | 812 | 812 | 812 | 812 | 812 | 812 |

**NRTI** – Nucleoside Reverse Transcriptase Inhibitor; **NNRTI** – Non-Nucleoside Reverse Transcriptase Inhibitor; **INSTI** – Integrase Strand Transfer Inhibitor; **PI** – Protease Inhibitor

**Table S3: Evolution of the proportion of participants with boosted regimen**

|  | 2017 | 2022 | Evolution : p-value of Mc Nemar test |
| --- | --- | --- | --- |
| Total number of participants | 812 | 812 |  |
| Number of participants with boosted regimen (Cobistitat or Ritonavir) | 262 (171 + 91) | 140 (121 + 19) | <0.0001 |
| % of the total * | 32.3 % | - 1. % |  |

**Table S4. Analysis of concomitant medications and drug-drug interactions (DDIs) in the subgroup of patients under follow-up in 2012 and 2016, as well as between 2017 and 2022.**

*^a^ Generalized Estimating Equations Model (GEE) ; ^b^ Linear mixed model ; ^c^ Linear model*

**Table S5: Non-ARV drugs implicated in DDIs, CBIP classification**

|  | | Year | | | | | |
| --- | --- | --- | --- | --- | --- | --- | --- |
|  |  | 2017 | 2018 | 2019 | 2020 | 2021 | 2022 |
| CBIP class | CBIP subclass | 2 | 6 | 4 | 3 | 4 | 3 |
| Dermatology | corticosteroid |  |  |  |  |  |  |
| Gynaecology- obstetrics |  | . | 1 | . | . | . | . |
| Ear-Nose-Throat | corticosteroid | 5 | 2 | 2 | 3 | 4 | 3 |
| Osteo-articular pathologies | NSAIDs | 1 | 1 | . | . | . | . |
| Haemostasis drugs | Antiaggregant drugs | 4 | 1 | 2 | 1 | 1 | . |
|  | Anticoagulant drugs | 1 | 1 | . | . | 5 | 4 |
| Cardio-vascular system | Calcium antagonist | 5 | 5 | 7 | 6 | 6 | 3 |
|  | Antiarrhythmic | 1 | 1 | . | . | . | . |
|  | lipid-lowering agent | 6 | 6 | 2 | 3 | 2 | 1 |
| Gastro-intestinal system | PPI | 6 | 3 | 3 | 5 | 7 | 5 |
|  | antiemetic | . | 1 | . | . | . | . |
|  | corticosteroid | . | 1 | . | . | . | . |
| Central nervous system | antidepressant - antipsychotic | . | . | 1 | 1 | 1 | . |
|  | antiepileptic | 1 | 4 | 1 | . | . | . |
|  | antipsychotic | 1 | . | . | . | . | . |
| Respiratory system | asthma and COPD | 9 | 5 | 8 | 5 | 6 | 4 |
| Vitamins, minerals and dietary supplements therapeutic class |  | 4 | 2 | 1 | . | . | . |
| Total number of DDIs | | 46 | 40 | 31 | 27 | 36 | 23 |

CBIP: Belgian pharmacotherapeutic information center
